# Breastfeeding as a balancing act – pregnant Swedish women’s voices on breastfeeding

**DOI:** 10.1186/s13006-020-00257-0

**Published:** 2020-03-05

**Authors:** Karin Cato, Sara M. Sylvén, Helena Wahlström Henriksson, Christine Rubertsson

**Affiliations:** 1 Department of Women’s and Children’s Health, Uppsala University Hospital, 751 85 Uppsala, Sweden; 2 Department of Neuroscience, Psychiatry, Uppsala University Hospital, 751 85 Uppsala, Sweden; 3 Center for Gender Research, |Uppsala University, Thunbergsvägen 3G Box 527, 75120 Uppsala, Sweden; 4 Department of Health Science, Faculty of Medicine, Lund University, Box 188, 22100 Lund, Sweden

**Keywords:** Breastfeeding, Breastfeeding support, Motherhood, Pregnancy, Qualitative

## Abstract

**Background:**

Breastfeeding provides health benefits to both women and children. The rationale behind an individual woman’s decision to breastfeed or not can depend on several factors, either independently or in combination. The aim of the current study was to explore attitudes towards breastfeeding among pregnant women in Sweden who intend to breastfeed.

**Methods:**

Eleven mothers-to-be, one of whom had previous breastfeeding experience, participated in the study. The women were interviewed either by telephone or face-to-face during late pregnancy, with the aim of exploring their attitudes towards breastfeeding. A semi-structured interview-guide was used, and the transcripts of the interviews were analyzed using thematic analysis. The social ecological model of health is the theory-based framework underpinning this study. The model provides a comprehensive approach to understanding the factors that influence breastfeeding intention.

**Results:**

When interviewed during pregnancy, women described breastfeeding as *a balancing act between societal norms and personal desires.* The women perceived a societal pressure to breastfeed, however it was accompanied by boundaries and mixed messages. This perceived pressure was balanced by their own knowledge of breastfeeding, in particular their knowledge of other women’s experience of breastfeeding. When envisioning their future breastfeeding, the women made uncertain and preliminary plans, and negotiated the benefits and drawbacks of breastfeeding. There was a wish for individual breastfeeding support and information.

**Conclusions:**

Pregnant Swedish women perceive their future breastfeeding as a balancing act between societal norms and personal desires. These findings suggest that while discussing breastfeeding during pregnancy, it could be of interest to collect information from pregnant women on their knowledge of breastfeeding and from where they have gained this knowledge, since stories from family and friends may make them question their own capacity to breastfeed. A thorough review of the woman’s experiences and attitudes of breastfeeding is important in order to offer the best evidence-based breastfeeding support.

**Trial registration:**

Ethical approval for the study was obtained from the Regional Ethical Review Board in Uppsala (Dnr: 2017/256).

## Background

Breastfeeding is widely known to provide health benefits for both mother and child [1]. Thus, it is recommended by the World Health Organization that women breastfeed exclusively for 6 months [2]. In Sweden, the recommendations are similar, although they include an amendment declaring that the introduction of “tiny tastes of solid food from the age of 4 months is harmless if it does not affect continuous breastfeeding for up to 1 year or longer [3]. These recommendations are distributed to families in Sweden by the National Food Agency and by healthcare services, for example during prenatal classes or at family healthcare centers. Statistics in Sweden, based on information collected in primary care, show that 93 % of all newborns are breastfed 1 week postpartum, of whom 78% are breastfed exclusively. By 6 months of age, the exclusive breastfeeding rate has plummeted to approximately 15% [4]. In Sweden, the WHO Code for Marketing of Breast-milk Substitutes was adopted in 1983 and The Baby-Friendly Hospital Initiative was introduced in 1993. By 1997, all maternity centers in Sweden were approved as baby-friendly, working according to the ten steps in a positive breastfeeding climate [5]. Since then, the Baby-Friendly Initiative’s presence in Sweden has declined due to the lack of a national organization overlooking these issues, and breastfeeding rates have subsequently decreased [4].

The vast majority of pregnant women in Sweden receive antenatal care from midwives during pregnancy. Women with uncomplicated pregnancies attend an antenatal clinic for check-ups approximately nine times before giving birth. At least two of these visits should address breastfeeding and each woman’s wish to breastfeed or not to breastfeed should be documented in her medical records. Furthermore, all women (and their partners) expecting their first child are invited to parental classes during pregnancy. In 2016, 71% of all nulliparous women in Sweden participated in antenatal classes [6].

Internationally, sociodemographic factors such as level of education and Body Mass Index have been reported to affect breastfeeding behavior [7, 8], but studies also point out that psychosocial factors, such as breastfeeding self-efficacy and breastfeeding expectations, are even more predictive of breastfeeding [9]. A common reason for the cessation of breastfeeding is the perception of not producing enough milk or experiencing primarily unexpected difficulties related to breastfeeding [10]. In a Swedish study, breastfeeding and, most importantly unsuccessful breastfeeding with related complications or difficulties that lead to breastfeeding cessation cause feelings of guilt and inner thoughts of being a bad mother [11].

According to international studies, decisions about and planning for breastfeeding are often made prior to birth [12, 13] and breastfeeding is often regarded as a personal choice, although it is influenced by maternal knowledge of the health benefits and a perceived pressure to make the “right choice” according to societal norms [13]. Knowledge of breastfeeding and its health benefits empowers women so it is important to offer both prenatal classes and individual discussion time on this issue [14].

Support from her partner is often highly valued by breastfeeding mothers, and the importance of a partner being positive towards breastfeeding manifests itself in a stronger intention to breastfeed and longer breastfeeding duration [15]. Mothers’ decisions on breastfeeding after birth are affected by prenatal opinions of the partner, her extended family, and healthcare providers [16].

Breastfeeding in social settings is an issue that has often been debated in the Swedish media. Women can experience negative comments when breastfeeding in public and are on occasion told to cover themselves. Internationally, breastfeeding in public has been described as a barrier to breastfeeding as it is not always viewed as acceptable [12, 13], and this may influence breastfeeding duration [17]. An American study, investigating whether women’s intentions to breastfeed were related to their comfort with breastfeeding in public settings, reported that women who felt uncomfortable breastfeeding in social settings more often had the intention of not breastfeeding exclusively [18].

Plans or intentions to breastfeed often depend on the working situation of the woman. In most countries, parental leave is short or non-existent [19], leaving the mother with the choice to either stay at home by giving up her job, or choosing the method of feeding her baby according to the parental leave possibilities and her job [20]. In Sweden, in general, women do not need to face this issue since the government grants parents 480 days of leave per child. Statistics from the Swedish Social Insurance Report show that most parental leave days are spent during the first 2 years of the child’s life and the majority of leave is used by the mother (76.3%) [21]. Hence, issues other than those related to work influence Swedish women when it comes to planning their future breastfeeding, as well as their choice of breastfeeding exclusively, partially, or not at all.

Breastfeeding intention is associated with breastfeeding exclusivity and duration. Most women in Sweden initiate breastfeeding according to the recommendations given to them. Despite this, breastfeeding rates in Sweden are declining, especially in the first few months postpartum. In 2015, 95% of newborns were breastfed at 1 week postpartum. This figure drops to 83% at 4 months and 63% at 6 months [4]. Attitudes towards breastfeeding and supplying breastfeeding support, as expressed by pregnant women who intend to breastfeed, are important to explore in order to gain a deeper understanding of women’s needs.

### Aim

To explore attitudes toward breastfeeding among Swedish pregnant women intending to breastfeed.

## Methods

### Participants and data collection

The study was conducted in Sweden and Swedish-speaking women intending to breastfeed were invited to participate. Respondents were recruited during parental classes at three maternity centers allocated in both urban and rural parts of Uppsala County during the period October to December 2017. The first and second author of the study held a short presentation giving details of the study for parental classes and distributed written information. A number of midwives approached women about participating in the study during regular visits. Posters presenting the study were put up in the waiting rooms of the maternity centers. Women interested in participation were given the study’s e-mail address. Some of the women had friends who were also interesting in participating, leading to three women being recruited via the snowball technique. Since the nature of recruiting participants involved informants contacting the first author for inclusion, we have no information about women who were not interested in participating. Participating women were told that their participation was voluntary and that they could withdraw their participation at any time. Further, participants were informed that all the data collected would be treated confidentially so that the identification of any particular woman would not be possible. After obtaining consent from the women, individual interviews were carried out by the first author, either face to face or by telephone depending on the woman’s choice. All interviews were conducted in Swedish and audio-recorded. The semi-structured interview guide designed by the authors included open-ended questions that allowed the women to discuss whatever they felt was important. After the initial questions on age, week of pregnancy, parity, education, work situation, and family status, the questions on breastfeeding commenced with: “Please explain what the word ‘breastfeeding’ means to you”. Other questions were formulated, such as: “What have you heard about breastfeeding?”, “How are you planning to feed your baby?”, and “What does breastfeeding support mean to you?”. To gain a deeper understanding, phrases such as “Please tell me more” and “Explain what you mean” were used. Two pilot interviews were conducted in the spring of 2017 to validate the wording of the questions used in the semi-structured interview guide. Only minor changes were needed after the first pilot interview and therefore the second pilot interview was included in the study. Except for this interview, all interviews were held during the period October 2017 to January 2018. The interviews lasted between 20 and 50 min with an average of 30 min. The informants chose the date and time of their interview, which led to all the women being at home for their interview, ten women were interviewed by telephone and one woman was interviewed face to face. No repeated interviews were held. All interviews were conducted, audio-recorded, and transcribed verbatim by the first author.

In total, ten nullipara women and one primiparous woman gave oral and written consent to participate in the study. The women’s ages ranged from 27 to 37 years, with an average age of 30.6 years. The interviews were held in pregnancy week 34–40 (average week 36), as this is the stage in which the antenatal midwife summarizes the pregnancy and the woman’s plans regarding breastfeeding. Nine of the women had university-level education. Nine were employed, one was between jobs, and one was studying. All participating women were cohabiting with the father of the expected baby and they lived in both rural and urban areas in Sweden. After interviewing the 11 women, saturation was reached, as similar answers were given. The social ecological model of health is the theory-based framework underpinning this study. The model provides a comprehensive approach to understanding the factors that influence breastfeeding intention, since it argues that influencing factors are the result of interaction between multiple factors, including both individual factors and societal factors [22].

### Data analysis

The analysis process was conducted following Braun & Clarke’s description of thematic analysis [23]. By using this method, the researchers identify, analyze, and report themes within data. It organizes and describes the data in detail and interprets various aspects of research topics. The social-ecological model of health is the theory-based framework underpinning this study [22]. The model provides a comprehensive approach in understanding the factors that influence breastfeeding. The analyses began while transcribing the interviews, when patterns of meaning were noticed. An initial understanding appeared by reading through each transcript multiple times. Initial codes were identified, and extracted into paper strips, and manually grouped into initial themes. The codes were regrouped several times, initially by the first author and later together with the whole research team. To find relationships between the themes, thematic maps were created. All codes and themes were compared and re-organized several times, until the authors agreed on the themes and the overarching theme of the study.

Two of the authors are midwives with clinical experience of postpartum care and breastfeeding support and one author is a physician, specializing in psychiatry with additional experience of obstetric care and breastfeeding support. The fourth author is a humanities/gender studies scholar, specialized in narratives about parenthood. While constructing the interview guide, and throughout the analysis, the authors brought their different disciplines and critical conventions to bear on formulations and structure, thereby paying attention to preunderstandings that could influence the analysis.

## Results

The aim of the study was to explore attitudes toward breastfeeding among pregnant Swedish women who intend to breastfeed. The overarching theme, *“Breastfeeding as a balancing act between societal norms and personal desires”,* consisted of two themes: “Conflicting societal norms stabilized by women’s knowledge” and “Envisioning breastfeeding”. Subthemes where defined within each theme.

### Conflicting societal norms stabilized by women’s knowledge

The theme *Conflicting societal norms stabilized by women’s knowledge* represents the women’s thoughts on a perceived pressure to breastfeed from society, yet one that was balanced by their own knowledge about breastfeeding. The sub themes within this theme were: *A perceived pressure to breastfeed, Social constraints on the breastfeeding body* and *Obtaining breastfeeding knowledge.*

### A perceived pressure to breastfeed

The women expressed that there is an underlying pressure from society to breastfeed, and if they do not succeed, they will be seen as an inferior mother. Some women described information on breastfeeding as propaganda about benefits, which leads to feelings of guilt if not succeeding with breastfeeding, and for some a feeling of being subjected to information that is not trustworthy.

*“Society thinks that mothers should breastfeed. For example, newspapers write that breastfeeding makes your child more intelligent*. *.*. *everyone wants an intelligent child, don’t they?*. *.*. *Most people want to give their baby the best and it’s a tough thing to hear when you can’t accomplish that.” (Respondent 1).*

*“All midwives I meet say it’s better with breast milk, it protects against allergies and so on, while other people say it doesn’t. It’s difficult to know what’s correct.” (Respondent 9).*

The women also described friends and relatives who had not succeeded in breastfeeding talking about feelings of shame and guilt. On the other hand, one woman with personal breastfeeding experience expressed feelings of guilt that she was able to breastfeed while others could not, as if she, when breastfeeding, was a “constant reminder of the failure of others”. It was further discussed that there are limits in society regarding perceptions that you should breastfeed; such as you should breastfeed but not for too long, i.e. not to breastfeed a child over 1 year of age.

*“You get criticized if you stop breastfeeding, as if you’re vain or lazy*. *.*. *, and if you do it for too long it’s regarded too hippie, so you should [breastfeed] a perfect length of time in between.” (Respondent 6).*

### Social constraints on the breastfeeding body

Some women perceived society as viewing the female body as sexualized in the context of breastfeeding. Some women mentioned that it could be problematic to breastfeed in public, although this was seen as an issue more common in countries other than Sweden.

*“Sometimes you hear that it’s not okay to breastfeed in public, that it’s dirty and that the breast is connected to sexuality” (Respondent 3).*

*“People find it annoying that women use their body to feed a baby” (Respondent 4).*

Some women thought that being criticized when breastfeeding in public was unfair, since breastfeeding was also regarded as a natural act, part of mothering a baby. The women’s opinion on the topic was to ignore any comments while breastfeeding in public since they perceived that their breastfeeding in public should not be anyone else’s concern.

*“If you need to do it, you kind of have to*. *.*. *it can’t wait. So if you have to do it, you just do it regardless.” (Respondent 7).*

### Obtaining breastfeeding knowledge

The women described different sources of breastfeeding information and knowledge. Some women also described a difference between information about breastfeeding given to the public in general and information addressed to pregnant women and their partners, meaning that during pregnancy breastfeeding information became more close to reality and more targeted towards them as expectant mothers. Two women reported that they had read scientific articles on the subject, but more commonly mentioned sources were media, internet, books, and parental classes. All women had shared thoughts of breastfeeding with their partner and gathered information on the subject through family, friends, and colleagues. Most stories of breastfeeding told by family, friends, and colleagues focused on breastfeeding problems. Although these stories often described breastfeeding as a challenge, including being in pain and suffering, the stories were regarded as positive by the women. According to the women, these stories gave comfort, hope, and time for reflection and preparation, that whatever happens will be okay and that the pressure of breastfeeding faded. Simultaneously, these stories were a reason to not take successful breastfeeding for granted.

*“I think it would have been harder if I didn’t know anything about breastfeeding problems. My friend struggled for several months and didn’t dare to ask for help, she felt ashamed*. *.*. *but I feel glad I talked to her and now know that breastfeeding can be difficult and that it’s okay even if it doesn’t work*. *.*. *I feel prepared.” (Respondent 5).*

All women were satisfied with the amount of time their midwife had given to discussing breastfeeding during their pregnancy. Nonetheless, breastfeeding information varied from short discussions to in-depth discussions. Some women stated that it was hard to discuss and take in information about breastfeeding during pregnancy and most women expressed a need to discuss various breastfeeding problems and their solutions as well as different feeding methods, especially since several women had the perception that breastfeeding would be a challenge due to experiences of others.

*“When will they [healthcare professionals] inform me about bottle feeding? Their main message is that breast milk is best for the baby*. *.*. *but do I really have to give it by breastfeeding? I could just as well give it [breast milk] by bottle feeding.” (Respondent 7).*

### Envisioning breastfeeding

The theme *Envisioning breastfeeding* and its subthemes *Uncertain plans* and *Negotiating benefits and obstacles* represent the participant’s perceptions of their future breastfeeding.

### Uncertain plans

When participants talked about their future breastfeeding, they all expressed a sincere wish to breastfeed. However, there were feelings of insecurity concerning their capability to succeed, and phrases such as. .. *if possible*. *.*. *if I manage, I hope it works* were often used*.*

*“I’ve always wanted to breastfeed if possible, but I have understood that it may not be that easy.” (Respondent 1).*

There was also a concern regarding breastfeeding duration; the women mentioned that it was difficult to speculate about how long they would continue breastfeeding, although they mentioned a willingness to breastfeed according to recommendations, i.e. 6 months. Some women stated that they would breastfeed as long as they themselves wanted, while others commented that the baby would decide when to stop or that time would tell.

*“I haven’t really thought about duration, but hopefully for at least six months so he [the baby] gets the best start.” (Respondent 8).*

### Negotiating benefits and obstacles

The women regarded breastfeeding as ‘snuggly’, an opportunity to be close to the baby. They also mentioned that breastfeeding is convenient and flexible, compared to handling bottles and preparing formula, which was regarded as bothersome. Breastfeeding was described as easy and flexible since the breast milk is always ready for the baby and available at all times. One woman explained that she would feel safer breastfeeding her baby, knowing that the baby would get all the nutrients needed. Since breastfeeding was seen as the most practical way to feed a baby, the women stated that they hoped they would succeed in breastfeeding.

*“If it works*. *.*. *then I see no point in dealing with formula and such, it [breastfeeding] feels like the most practical alternative, it has everything that’s needed.” (Respondent 9).*

The women mentioned various positive health benefits associated with breastfeeding and that breastfeeding facilitates bonding and attachment between mother and baby. Some women declared that such breastfeeding benefits were crucial for them and were their main reason for wanting to breastfeed.

*“I want to breastfeed because it enhances bonding*. *.*. *and it’s healthy for the baby. Those kind of things make it important to me.” (Respondent 10).*

Most women disclosed that their partners were positive towards breastfeeding and that it was seen as the woman’s choice whether or not to breastfeed, a choice that was generally respected by the partner. Some women disclosed that this choice was made together with their partner. Nevertheless, breastfeeding was mentioned by some women as a possible obstacle to the father’s ability to bond with the baby. Some women stated that they perhaps would want their partner to bottle-feed the baby part time, even if breastfeeding was successful. It was mentioned that it could be good to share feeding the baby with the partner early on and that shared feeding was a way of supporting mothers, since it would give them time to sleep at night or time on their own. Some participants saw shared feeding as a way to gain equality between parents.

*“We want to use the bottle early on, so he [my partner] can bond with the baby and get a moment of snuggling.” (Respondent 4).*

The initiation of breastfeeding was envisioned as a troublesome learning period, which could determine or define the success or failure of the whole breastfeeding experience. Breastfeeding was seen as a challenge in terms of it maybe being painful and time consuming.

*“It may hurt and be bothersome. I’m somewhat prepared for that, that it won’t work from day one. I think that it will be troublesome*. *.*. *for at least a few weeks.” (Respondent 10).*

A concern, and awareness, among the participants was facing potential breastfeeding problems, which were described as possible obstacles to continued breastfeeding, especially if the problems start early on. Breastfeeding problems were often described in the context of psychological wellbeing where the wellbeing of the woman was seen as more important than breastfeeding at any costs.

*“Time will tell if I continue breastfeeding with sore nipples*. *.*. *I want to feel happy with my baby.” (Respondent 4).*

A difficult start to breastfeeding or other breastfeeding problems such as mastitis were expressed as psychological strains, especially when accompanied by feelings of failure. Several women expressed that cessation of breastfeeding due to problems would hopefully not cause feelings of failure, but they expressed concern about this.

*“If I’m not capable of breastfeeding I don’t want to feel like a failure.” (Respondent 5).*

If they were to face breastfeeding problems, which the women perceived as most common during the initiation phase, they expressed wishes of individualized support, given calmly by someone with experience and knowledge, who did not necessarily have to be a healthcare professional.

*“I think it [breastfeeding support] should be given by a gentle, warm and calm person who can show me how it’s done with [their] body language, voice, and whole personality.” (Respondent 1).*

The women expressed the importance of someone listening to them and giving individual support. Initiation of breastfeeding was seen as the time where most breastfeeding support would be needed to make breastfeeding successful from the beginning. Some women stated that the term “It looks fine”, which is perceived to be commonly mentioned by healthcare professionals while observing breastfeeding, should instead be phrased as a question i.e. “How does it feel?”, to ensure that the breastfeeding observation would lead to a meaningful counselling session.

*“I don’t want it to look fine, I want it to feel fine.” (Respondent 7).*

Although prepared for a troublesome initiation, the women looked forward to experiencing the perceived benefits of breastfeeding such as cuddling and feeling close to the baby.

*“I know that it can be complicated and hurt and that you may get sore nipples in the beginning. It can be troublesome*. *.*. *but also snuggly” (Respondent 11).*

## Discussion

### Results discussion

Since breastfeeding is presented by both healthcare professionals and society as being the best option, women described a societal norm or pressure to breastfeed in order to be regarded as a “good mother”. This link between breastfeeding and good mothering, and feelings of guilt if not succeeding in giving the baby “the best” has been described previously [24, 25]. In a Swedish context, feelings of guilt and thoughts of being a bad mother were also reported by Palmer et al. [11]. The complexity of shame caused by breastfeeding, as described by the primipara participant included in the study due to feeling responsible for the perceived failure of others when breastfeeding in public, has been found to exist among mothers who breastfeed as well as among non-breastfeeding mothers when feeding in a social context [26]. A perception of guilt, and a need to defend their choice of formula feeding was suggested by some of the women in the present study. It has been suggested that different feeding methods should be addressed by healthcare professionals to provide a more balanced view, so mothers who choose formula feeding avoid criticism [27]. However, this creates a dilemma since breastfeeding, rather than formula feeding, should be supported by healthcare professionals due to the benefits it beings to maternal and infant health [1, 2]. Further, since not being able to breastfeed brings out feelings of shame and guilt and therefore affects the emotional well-being of mothers, the challenges of exclusive breastfeeding need to be addressed and recognized [28].

Although the study participants stated that they were satisfied with the amount of information they received, this does not necessarily mean that they received all the information they actually needed. Most women reported receiving information on breastfeeding from parental classes, including information on breastfeeding health benefits, which were said to be both crucial in choosing to breastfeed as well as problematic as they could be a psychological burden if not successful. Nevertheless, the women reported other aspects of breastfeeding to be important in their choice as well, such as breastfeeding being practical and available at all times. As suggested by Losch et al., already in 1995 [29], it could be of importance to increase information provided to expectant parents on practical aspects of breastfeeding and give strategies for decreasing breastfeeding problems, as breastfeeding duration and exclusivity increases by support offered in antenatal care [30] .

A recent study shows that although the media presents a wide range of topics about breastfeeding, factors that facilitate breastfeeding are rarely covered and the focus is on barriers to breastfeeding [31]. It is unclear if this is the case in Swedish media, but it could indeed be one of the reasons why the participants perceived breastfeeding as troublesome. Some women reported that negative experiences from others might give them comfort and support and reduce loneliness if they were to experience breastfeeding problems. Loneliness in motherhood might arise from self-comparison with the perceived societal norms on mothering, as suggested by Lee et al. [32]. Another study of 191 Canadian women concluded that mothers need information on breastfeeding’s common challenges and solutions, and how to overcome these through individualized support that meets psychosocial and emotional needs; hence acknowledge both positive and negative breastfeeding experiences [33].

Most participants had discussed breastfeeding with their partner and mentioned him as an important support in their future breastfeeding and some envisioned him taking care of household duties while she would breastfeed. All of them perceived their partner as positive towards breastfeeding. Partner support is crucial and a discussion on the topic, initiated by healthcare professionals, preferably should be held both prenatal and postpartum with both partners present [34]. Some women expressed beliefs about the importance of sharing feeding with the partner for the partners’ ability to bond with the baby and as a way to gain equality between the parents. However, infants are less likely to be breastfed when the mother’s partner does not use parental leave days during the infant’s first year, which implicates that shared parenting, rather than feeding mode, is associated with longer breastfeeding duration [35].

All the women reported feelings of insecurity with regards to their capability to breastfeed and concern about the initial stage of breastfeeding. A Swedish study comparing mothers’ experiences of breastfeeding over a decade showed that nowadays women tend to experience breastfeeding to be more difficult than before and also reported higher levels of insecurity [36]. It is possible that stories of breastfeeding problems from friends and family might have caused this, and as mentioned above, the media might also be an influence. Nevertheless, if problems related to breastfeeding was not spoken of, women might get an unrealistic image of breastfeeding that could lead to negative emotions [37].

Most women thought that the support given directly after birth would be crucial for their breastfeeding experience. Since they perceived initiating breastfeeding to be a challenge, they wanted adequate support from healthcare professionals. Indeed, the initiation phase of breastfeeding is of great importance for breastfeeding success and adequate breastfeeding support may be crucial [38, 39].

By applying the social ecological model of health to the findings, which considers the interplay of multiple levels of a social system and interactions between the individual and the environment, one can envision pregnant women being influenced by both micro-level and macro-level factors. The social ecological model of health consists of the individual level, the interpersonal level, the community level, the organizational level, the policy level, and the interaction between these multi-factorial levels. Applicable to the present study, on the interpersonal level, stories from friends and family influenced the way in which the participating pregnant women thought about breastfeeding. Further, pregnant women can perceive a pressure from society to breastfeed, including feelings of receiving mixed messages about breastfeeding, and an uncertainty regarding their capability to breastfeed. To better meet the needs of women who intend to breastfeed, healthcare professionals should consider each woman’s individual needs and be aware of the multi-factorial connections of environmental influences and the social relationships that might have an impact on them. The findings from this study identify opportunities to inform and support women in the prenatal period in an effort to support breastfeeding.

### Methodological considerations

Due to the recruitment method used, the participants in this study may represent women who are more confident in their decision to breastfeed or more interested in breastfeeding than other women, hence factors important to women who were less confident about or less interested in breastfeeding might be missing, which could influence the results. However, we were interested in women who intended to breastfeed. As with many other studies, the present study engaged highly educated women, which is a group well known for breastfeeding for a longer duration.

Further, one could question the timing of the interviews, as some of the women had not yet received much information on breastfeeding from their midwife or attended parental classes while others had, and information about breastfeeding is known to enhance the rate of choosing to breastfeed over bottle or mixed feeding with formula [30]. Nevertheless, all the women intended to breastfeed their coming newborn in the future. Only women able to communicate in Swedish were invited to participate in the study, which must be taken into account when interpreting the findings. Most interviews were conducted by telephone, which could affect the results since nonverbal actions were not observed. Interviews held by telephone may, on the other hand, give respondents more confidence to share sensitive information [40].

Establishing credibility and confirmability is important when conducting qualitative studies [41, 42]. Credibility was increased by the research team consisting of a collaboration of professionals with backgrounds in medicine and the humanities, which yielded different opinions in designing the question guide as well as during the analysis process. Initial themes and subthemes were identified by the first author. Confirmability of the study was enhanced through the research groups’ awareness of their pre-understanding of the subject.

## Conclusion

When interviewed during pregnancy, women describe breastfeeding as a balancing act between societal norms, personal concerns regarding ability, and acquired knowledge. The women requested more information and in-depth discussions about breastfeeding and potential breastfeeding problems during pregnancy and wished for individualized support during the initiation of breastfeeding. While discussing breastfeeding during pregnancy it could be of interest to collect information from pregnant women on their knowledge of breastfeeding and from where they have retrieved this knowledge, since stories from family and friends may make her question her own ability to breastfeed. A thorough review of the woman’s experiences of and attitudes towards breastfeeding is important in order to offer her the best evidence-based breastfeeding support.

## Supplementary information

**Additional file 1 Supplementary figure.** Example of coding.

## Data Availability

Due to the small study size, in order to safeguard the integrity of the respondent’s data sharing is not applicable.
