# Supplementary material for: Breastfeeding as a balancing act – pregnant Swedish women’s voices on breastfeeding
Source: Int Breastfeed J. 2020 Mar 5;15:16. doi: 10.1186/s13006-020-00257-0 (PMC7059277; doi:10.1186/s13006-020-00257-0)
Supplement: Supplementary file 1 — Additional file 1 Supplementary figure. Example of coding. [file 13006_2020_257_MOESM1_ESM.docx]

| **Overarching theme** | **Themes** | **Subthemes** | **Subtheme description** | **Example qouatation** | **Example of codes** |
| --- | --- | --- | --- | --- | --- |
| Breastfeeding as a balancing act between societal norms and personal desires | Conflicting societal norms stabilized by women’s knowledge | A perceived pressure to breastfeed | The women expressed that there is an underlying pressure to breastfeed, but simultaneously that there are many rules on how, where and when to do it or not to do it | “ You get criticized if you stop breastfeeding, as if you’re vain or lazy…and if you do it for too long it’s regarded hippie, so you should do it a perfect length of time in between” | Pressure, underlying rules, mixed messages |
|  |  | Social constraints on the breastfeeding body | Some women perceived society as sexualized regarding the female body in the context of breastfeeding, thus making it problematic breastfeeding I public places. | “Sometimes you hear that it’s not okay to breastfeed in public, that it’s dirty and that the breast is connected to sexuality” | Sexualization of breastfeeding/of the breasts |
|  |  | Obtaining breastfeeding knowledge | The women described different sources of breastfeeding information and knowledge | “My friend struggled for several months and didn’t dare to ask for help, she felt ashamed…but I feel glad I talked to her and now have knowledge that breastfeeding can be difficult…” | Stories from friends and family, parental class, information from health visits |
|  | Envisioning breastfeeding | Uncertain plans | All women expressed a wish to breastfeed, but spoke of insecurity concerning their capability to succeed. | “ I always wanted to breastfeed if possible, but I have understood that it may not be that easy” | A wish to breastfeed, insecurity |
|  |  | Negotiating benefits and obstacles | The women discussed different benefits and obstacles regarding breastfeeding, important to them | “ I know that it can be complicated and hurt and that you may get sore nipples in the beginning. It can be troublesome…but also snuggly” | Benefits of breastfeeding, risk of breastfeeding problems |
|  |  |  |  |  |  |
|  |  |  |  |  |  |

**Supplementary figure.** *Example of coding*
